# Retraction notice to “Knowledge and attitude toward cervical cancer screening among women aged 30–49 years attending selected health facilities in Ethiopia: using structural equation model” [Heliyon 10 (2024) e31596]

**DOI:** 10.1016/j.heliyon.2025.e43406

**Published:** 2025-05-22

This article has been retracted: please see Elsevier Policy on Article Withdrawal (https://www.elsevier.com/about/our-business/policies/article-withdrawal).

This article has been retracted at the request of the Editor-in-Chief.

Dr. Sara Debebe Jimma notified the Editor-in-Chief that the paper was published without her authorisation while also claiming sole authorship of the work.

The Editor-in-Chief contacted the corresponding author, who acknowledged that the work was conducted using data collected by Dr. Debebe and admitted to publishing the article without proper acknowledgment of the project team that sponsored the work. Efforts to obtain more information regarding this dispute and the work sponsorship by contacting the involved institutions have gone unanswered.

Consequently, the decision was made to retract the paper, with all authors agreeing to this action.

The scientific community takes a very strong view on this matter and apologies are offered to readers of the journal that this was not detected during the submission process.

